# Lymphadenectomy in the treatment of sarcomas – indications and technique

**DOI:** 10.3389/or.2024.1413734

**Published:** 2024-12-05

**Authors:** Piotr Dunaj, Ewa Żukowska, Anna M. Czarnecka, Maria Krotewicz, Aneta Borkowska, Paulina Chmiel, Tomasz Świtaj, Piotr Rutkowski

**Affiliations:** ^1^ Department of Soft Tissue/Bone Sarcoma and Melanoma, Maria Sklodowska-Curie National Research Institute of Oncology, Warsaw, Poland; ^2^ Faculty of Medicine, Medical University of Warsaw, Warsaw, Poland

**Keywords:** lymphadenectomy, sarcoma, metastasis, treatment, lymph node, surgery

## Abstract

Sarcomas are a rare type of malignancy with limited treatment options so far. This analysis aimed to describe the impact of lymphadenectomy on treating sarcoma patients. Sarcomas characterized by lymphatic spread are rare. For this reason, lymphadenectomy is not a procedure that is performed frequently. However, there are histological subtypes that spread more frequently through lymphatic vessels, such as rhabdomyosarcoma (RMS), epithelioid sarcoma (ES), clear cell sarcoma (CCS), and angiosarcoma. On the other hand, synovial sarcoma (SS) is not characterized by an increased tendency to lymphogenous metastases. In our study, we focus on these subtypes of sarcomas. The relationship between lymphadenectomy results and the subsequent prognosis of the patients was investigated. Metastases in the lymph nodes are diagnosed synchronously with distant metastases or when the primary tumor is detected. At the same time, despite lymphadenectomy, sarcoma patients developed further distant metastases. Currently, lymphadenectomy is not a routinely recommended method of treatment for patients with sarcomas. Most often, its potential use is indicated in the case of epithelioid sarcoma, clear cell sarcoma, and rhabdomyosarcoma after a previous positive sentinel lymph node biopsy (SLNB) result. Multicenter randomized prospective clinical trials on the role of lymphadenectomy in the treatment of sarcomas are needed.

## 1 Introduction

Patients with soft tissue sarcomas (STS) diagnosed in the early stages of the disease have a good prognosis. The possibility of using surgical cytoreduction methods in them prevents systemic dissemination of sarcoma cells (1). The 5-year relative survival rate ranges from 15% in the presence of distant metastases, to 56% in the presence of dissemination in regional lymph nodes and ends with 80% for localized forms detected in an early stage of development in the study population of STS patients with various histological subtypes (2). About half of patients with sarcoma will eventually reach an advanced stage of the disease with the presence of distant metastases (3). As a result, the median overall survival in the analyzed population of patients with disseminated STS (of various histological subtypes) is 12–18 months (4). The development of sarcomas in the initial phase is often clinically silent. This results in the correct diagnosis in an advanced stage of metaststic disease (5). Basile et al. showed that patients with STS of the extremities with metastases only in regional lymph nodes have a 5-year overall survival (OS) of 57.3% compared to a 5-year OS of 14.6% for cases with only distant metastases or a 5-year OS reaching 0% in patients with secondary foci of nodal and organs (6). The advanced stage of the development of sarcoma limits the use of surgical methods to palliative care. In such a situation, the primary goal is not to cure the sarcoma, but to ensure the best quality of the final phase of life. An example is the dissection of enlarged cervical lymph nodes that interfere with the patency of the respiratory or digestive tract (7).

Complete sarcoma resection is crucial for the patient’s prognosis (8). Metastases may be located at organ locations corresponding to the characteristic of the dissemination route of a given STS subtype. Therefore, radical resection of the primary lesion is then complemented, for example, by dissection of the locoregional lymph nodes or resection of individual pulmonary secondary sarcoma foci (9, 10). Resection of the malignant tumor in this group of patients provides a statistically significant improvement in their prognosis. Blay et al. show that people who undergo surgical cytoreduction of sarcomas (of various histological subtypes) by experienced physicians in this field achieve the 40-month overall survival in approximately 80% of almost 10,000 analyzed patients. Furthermore, the 40-month local relapse-free survival (LRFS) in the above study group is approximately 70% (11). Currently, special attention is paid to the radicality of the resection performed. Bilgeri et al. showed that 40-month OS characterizes patients with sarcomas (histologically differentiated) undergoing R0 resection in approximately 72% of them. In turn, 40-month LRFS was achieved by 85% of patients analyzed in this study (12). The development of methods to identify sarcoma cells favors the diagnosis of this disease and radical resections. In the case of patients with retroperitoneal sarcomas, an increase in R0 resections of up to 90% has been observed in recent years (13, 14) and improves the prognosis of these patients.

One of the ways to treat may be to assess the effectiveness of extrapolated therapeutic methods from other nosological units. This approach is used to treat rare malignant tumors, which are malignant tumors of the connective tissue (15). Sentinel lymph node biopsy (SLNB) or lymphadenectomy (lymph node dissection; LND) with good therapeutic effects are performed during radical resections of malignant tumors, such as breast cancer or melanoma (16, 17). However, the validity of lymph node dissection in patients with sarcomas is rarely described in the available literature. Sarcomas metastasize mainly through the bloodstream, but there are histological subtypes that have a predilection for lymphogenic dissemination. Patients with sarcomas with lymphogenic predilection for metastasis could benefit therapeutically from early SLNB or LND. Just as in the case of patients with gastric or renal cancer (18, 19). Therefore, SLNB or LND as components of cytoreductive surgical procedures could be used in specific histological subtypes of sarcomas. This publication aims to determine the role of LND in treating selected sarcomas based on a review of the available literature. The importance of the preceding SLNB was also considered.

## 2 Lymphogenic spread of sarcomas

The predilection of sarcoma to lymphogenous neoplastic dissemination is the starting point for considering lymphadenectomy as part of diagnostic and therapeutic procedures. In the entire population of people with soft tissue sarcomas of various histological subtypes, secondary lesions in the regional lymph nodes are rare. It occurs only in about 1% of these patients (20). However, early detection of secondary sarcoma lesions only in locoregional lymph nodes (N1) provides a chance for therapeutically effective malignant tumour resection (1). Detection of sarcoma in nonregional lymph nodes confirms M1 disease (21, 22). Secondary foci formed in this way make it impossible to carry out effective cytoreductive treatment and ultimately disturb the homeostasis of the system, leading to the death of the patient. Analysis of the STS patient database in the limb by Garcia-Ortega et al. indicates a poor prognosis for patients with metastases only to lymph nodes (OS = 21 months) and those with distant organ metastases (OS = 18 months). It was emphasized that patients with secondary foci simultaneously in lymph nodes and other organs have a shorter OS = 15 months. The authors indicated that lymph node metastases are unfavourable prognostic factors for OS and event-free survival (EFS) (23). Research by Emori et al. also confirms this observation. Patients with secondary nodal foci of sarcomas of various histological subtypes detected within 8 months after the diagnosis of the primary focus have a 5-year survival rate of 19% (24). The appropriate use of surgical oncological methods (including SLNB and LND) could improve the survival rates of these patients.

Reliable determination of the frequency of dissemination of sarcoma requires the use of appropriate diagnostic methods. The most reliable confirmation of secondary foci is detecting sarcoma cells in the histopathological material of the lymph node (25–27). However, palpation and modern imaging techniques do not allow an unequivocal diagnosis of a malignant neoplasm. Reliable evidence of a metastasis in a lymph node is its complete excision, followed by histopathological analysis. Wagner et al. showed the diagnostic advantage of sentinel lymph node biopsy over PET-CT (positron emission tomography-computed tomography) in detecting cancer cells of the sarcoma in the surrounding lymphoid tissue (28). Positron emission tomography imaging was characterized by only 57% sensitivity and 52% specificity. However, this procedure has also produced false negative results. Neville et al. showed that 17% of the regional nodal basins analyzed that were considered sarcoma-free had micrometastases (29). In addition, Wright et al. reported that approximately 5 of 100 patients with sarcoma had a false negative SLNB result (30). To increase the efficiency of intraoperative detection of neoplastic cells in lymph nodes, new techniques of biopsy analysis are proposed. Namba et al. indicate the advantage of the One-Step Nucleic Acid Amplification (OSNA) method over a typical histopathological examination performed by a pathologist. OSNA analyzes the mRNA present in the biopsy, which is performed by an appropriate analyzer, and the quickly obtained result is more reliable than the microscopic evaluation of the tissue (31). As such, more precise diagnostic techniques are being researched. It is proposed to use fluorescently labeled indicators specific for specific cancer tissues (32), or to analyze the concentration of cancer DNA (ctDNA) in the bloodstream (33).

Before surgical collection of lymph nodes for histopathological examination, suspicious oncological lesions may be initially identified in the patient’s history and physical examination. The available data confirms several factors responsible for the predilection of sarcomas to lymph node metastases. The presence of metastases to lymph nodes of sarcoma has been demonstrated most often when the primary tumor is located in the chest (5% of cases) or abdominal cavity (5% of cases). In the case of extremity sarcoma, secondary foci were found in 2% of patients (34). The predilection of STS perineal and rectal tissues reaching about 50% for metastasis to regional lymph nodes is underlined (35). Miccio et al. demonstrated that grade 3 of one of the selected histological STS subtypes, clear cell sarcoma, angiosarcoma, rhabdomyosarcoma, and epithelioid sarcoma, is associated with a risk of metastasis to the lymph nodes of approximately 12% (36). At the same time, Liu et al. reviewed data from more than 3,000 sarcoma patients who underwent regional lymph node biopsy and 73% of cases with lymph node involvement had a primary tumor of the head, neck or extremity and the patients had a median age of 24 years. On the contrary, the median age of patients with negative lymph node biopsies was 54 years. Rhabdomyosarcoma, clear cell sarcoma, epithelioid sarcoma, primary tumor size above 4 cm, location in the head and neck region, high-grade III and IV, male sex were also found to be risk factors for lymph node metastases (37). Liu et al., in a study on how to perform surgical treatment in patients with early-stage uterine sarcoma, do not recommend routine lymphadenectomy (38). This is due to the low probability of metastases to the lymph nodes, and when they are removed, the surrounding tissues are damaged, which complicates the course of the surgical procedure. Considering the primary tumor biopsy results and the above risk factors, a comprehensive assessment of the patient’s clinical condition allows us to avoid excessive resection, including LND.

**FIGURE 1:**
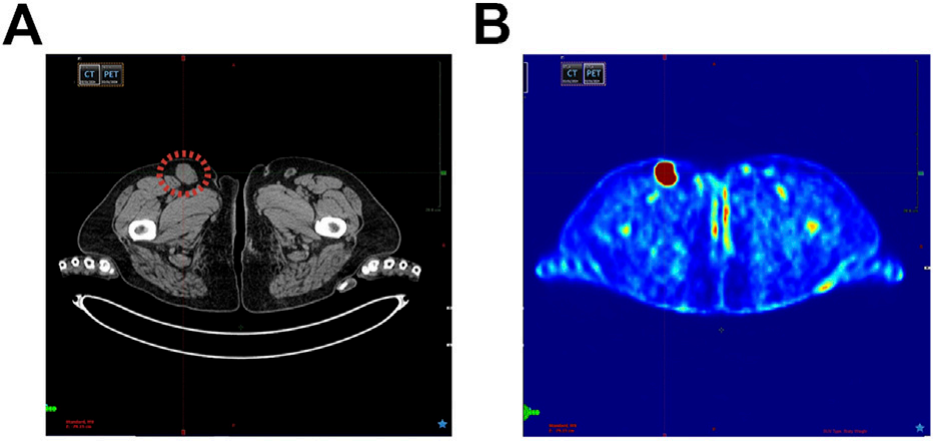
**(A)** Metastatic groin lymph node (marked in red) in a patient diagnosed with soft tissue sarcoma (STS), computed tomography (CT) image. **(B)** The same patient, lymph node metastases shown by positron emission tomography-computed tomography scan (PET-CT).

Imaging tests, e.g., CT (computed tomography), MRI (magnetic resonance imaging), PET-CT, and SPECT (single-photon emission computed tomography), help identify pathological changes of the nodal and extranodal region. In the ESMO (European Society for Medical Oncology) clinical practice guidelines on sarcomas, Gronchi et al. recommend CT-MRI to detect potential lymph node metastases (39). In the case of histopathological confirmation of a sarcoma subtype characterized by an increased probability of metastasis to the lymph nodes, PET-CT is recommended (Figure 1) (40). However, they do not allow for an unequivocal statement that the abnormality observed is a neoplastic metastasis. Mabuchi et al. demonstrated statistically significant false positive results when detecting secondary pelvic and paraaortic lymph node lesions in patients with malignant gynecological tumors using 18F-FDG-PET-CT (Fluorine-18 fluorodeoxyglucose PET-CT) (41). Hypermetabolic inflammatory foci in the lymph nodes gave the impression of metastases. Researchers suggest the possibility of differentiating the premetastatic niche from the true metastasis in the lymph node using SPECT using antibodies against the S100A8/S100A9 niche proteins. The increased expression of these molecules is responsible for the increased uptake of 18F-FDG, resulting in false positive PET-CT results (41).

The diagnosis and surgical treatment of primary sarcomas is well described in the literature. However, the lack of uniform guidelines for diagnostic and therapeutic procedures for secondary sarcoma lesions in the lymph nodes results in various procedures described in the few publications on this topic (42). Therefore, the possibility of presenting a reliable incidence of lymph node metastasis of specific sarcoma subtypes is limited. In the available literature, there are differences in the methodology to determine the presence of STS metastases to lymph nodes. This is because the authors of various publications use noninvasive imaging diagnostic methods or no specific technique is indicated (Table 1). On the contrary, few publications specifically indicate that the frequency of STS metastases to the SLN was determined based on the results of the SLNB (Table 2). Therefore, the range of the estimated frequency of lymphogenic STS metastasis varies in publications. Furthermore, the analyzes cover small groups of patients with a given rare subtype of rare diseases, such as sarcomas. This results in a limitation of the statistical significance of the presented results.

**TABLE 1:** Frequency of lymph node metastases in selected sarcoma subtypes.

Pathological type	Lymph node involvement (%)	References
Rhabdomyosarcoma	26–60	(43, 44,47, 48, 49)
Epithelioid sarcoma	7.5–18	(59, 154, 155)
Clear cell sarcoma	31–44	(69, 70, 71)
Angiosarcoma	1–23	(45, 60, 78, 79)
Synovial sarcoma	4.2–10	(89, 90, 91)

**TABLE 2:** Positive SLNB frequency in selected sarcoma subtypes.

Pathological type	Positive/total SLNB (%)	References
Rhabdomyosarcoma	0–50	(29, 30, 54, 67, 68, 156, 157, 158)
Epithelioid sarcoma	0–10	(30, 67, 68, 142, 156)
Clear cell sarcoma	0–50	(30, 67, 142, 156)
Angiosarcoma	0–5	(159, 160)
Synovial sarcoma	0–6	(30, 67, 142, 156)

### 2.1 Rhabdomyosarcoma (RMS)

RMS tends to metastasize through lymphatic vessels compared to other histological subtypes of STS (43). About 20% of patients with RMS show lymphogenic dissemination. The frequency of secondary foci in the lymph nodes is over 50% in pediatric patients with RMS (43–45). The location of this type of primary tumor in the region of the limbs and the genitourinary tract is associated with a particularly increased predilection for lymphatic metastases of 12%–24% (45, 46). Among 197 children with limb rhabdomyosarcoma, the presence of lymph node metastases was found in 33% (65 patients), and distant organ metastases in 32% (63 patients) (47). In head and neck RMS, local lymph node metastases were reported in 26% (43/165) patients (48). Among 109 patients with metastatic rhabdomyosarcoma, 60% of the cases had regional lymph node metastases (49). Failure of the applied RMS treatment is often due to locoregional recurrence of the sarcoma. Two-thirds of them are detected in lymph nodes that receive lymph from the area of the primary location of the sarcoma (50). The presence of RMS metastases in the lymph nodes is an unfavorable prognostic factor (51). The 5-year OS in RMS patients with regional lymph node (N1) metastases is 60%–65% (47, 52). Furthermore, Rodeberg et al. showed that patients with alveolar RMS have a statistically significantly worse prognosis in the case of locoregional lymph node involvement (N1M0) than patients with alveolar RMS N0M0 (53). The 5-year OS for the mentioned groups of patients was 46% and 80%, respectively. In patients with alveolar RMS, the prognosis is unfavorable when only locoregional lymph nodes are involved (N1M0) and comparable to patients with a single focus of distant organ metastases (M1). However, in patients with embryonal RMS, there were no statistically significant differences in prognosis in the presence or absence of secondary sarcoma lesions in locoregional lymph nodes (53). Therefore, the potential use of lymph node dissection as part of diagnostic and therapeutic procedures in patients with RMS seems reasonable, especially in pediatric patients (43, 54, 55). RMS is distinguished from other STS by its predilection for lymphatic dissemination. However, the hematologic route is a common means of metastasis in this sarcoma subtype (56).

### 2.2 Epithelioid sarcoma (ES)

**FIGURE 2:**
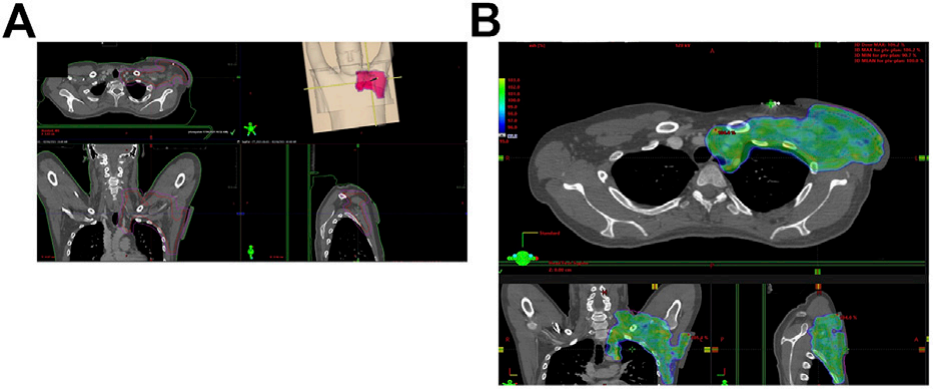
**(A)** Lymph node metastases in a patient with epithelioid sarcoma (ES). **(B)** Postoperative radiotherapy for metastatic axillary lymph nodes (2 Gy–60 Gy).

ES is the most common STS of the hand and wrist (57). However, it accounts for less than 1% of all soft tissue sarcomas. The median age of diagnosis of ES is 35 years and they are mostly men. It is characterized by a tendency to local recurrences, metastases to locoregional lymph nodes, and other organs, e.g., the lungs and brain. Numerous relapses can result from the spread of neoplastic cells along the fascia and tendons, which are difficult to access for effective cytoreduction (58). ES is distinguished from other STS by its high lymphatic dissemination rate. As shown by Kashyap et al. 65% of ES patients have metastases in the lymph nodes (59), resulting in a 5-year OS of approximately 50% (58, 60, 61). Other publications indicate that secondary lymph node foci of epithelioid sarcoma are present in 13%–32% of patients with this sarcoma subtype (20, 62, 63). In their analysis of patients with ES, Visscher et al. didn’t confirm a statistically significant unfavorable prognosis in those with N1 compared to those with N0 (64). They noted that locoregional lymph node metastases were detected at a median time of 7 months after the diagnosis of ES. Later, distant organ metastases were found in patients with N1. The investigators suggested that secondary nodal foci in patients with ES is a manifestation of the development of regional sarcoma rather than systemic dissemination of the disease, as suspected by (65). Moreover, Brady et al. in a retrospective cohort study of 1,550 patients with various subtypes of sarcomas showed that patients with epithelioid sarcomas represented 4% of all patients with sarcomas (64 of 1,550 patients) (66). The 5-year OS in the entire group of patients with this particular subtype of sarcoma was 87%, those without sarcoma cells in SLNB had a 5-year OS of 90%, and in patients with identified foci of epithelioid sarcoma in SLNB it was 43%. The 10-year OS in the individual analyzed patient groups was 80%, 82%, and 0%, respectively (66). In turn, in the ESMO clinical practice guidelines on sarcomas, Gronchi et al. indicate a less than 1% incidence of metastases in regional lymph nodes in the case of ES (39). However, they emphasize that the exceptions with a higher probability of finding lymphogenous dissemination are epithelioid sarcoma, synovial sarcoma, angiosarcoma, and clear cell sarcoma. In their case, CT-MRI is recommended to detect possible metastases in the lymph nodes (Figure 2) to avoid premature surgical SLNB, which carries the risk of possible side effects. Wright et al. in a meta-analysis showed that none of the 17 people with epithelioid sarcoma who underwent SLNB had sarcoma cells detected in biopsy (30). Similar results were found in two other retrospective studies of a small group of ES patients who underwent SLNB (67, 68). Therefore, there is potential to improve the prognosis of patients with ES by performing diagnostic SLNB and cytoreductive LND. However, a thorough radiological diagnosis is recommended first.

### 2.3 Clear cell sarcoma (CCS)

CCS has a high tendency to metastasize to regional lymph nodes, with approximately 25%–50% of patients showing the presence of lymph node metastases (69–71). In patients with CCS after SLNB, Wright et al. showed the presence of secondary CCS lesions among 35% (6/17) of them (30). Also, Andreou et al. in a similar patient population obtained 50% (6/12) positive SLNB results (67). In addition, Clark et al. analyzed the frequency of recurrence of CCS and metastases detection after resection of the primary tumor in 35 patients (72). Of these, 23% had local recurrence or metastasis in transit with a median of 9 months (range 2–79 months). On the contrary, neoplastic cells were found in the lymph nodes or distant organs in 63% of subjects at a median of 14 months (range 0–177 months) after resection of the primary tumor (72). The prognosis is poor due to the high tendency to local and distant metastases (73). Hocar et al., in an article analyzing the course of the disease in 52 patients with clear cell sarcoma, indicate that this sarcoma has a high risk to metastasize to regional lymph nodes as well as distant organs and a tendency to local recurrence (70). In this sense, it differs from typical sarcomas and resembles melanoma, hence the alternative name for CCS, soft tissue melanoma (70). In the study cited, 31% (16 patients) of the patients showed the presence of neoplastic cells in the lymph nodes. Distant metastases occurred in 56% (29 patients) cases (70). The 5-year OS in patients with CCS N1M0 is approximately 27%. In turn, in patients with CCS N1M1, the 5-year OS is 0% and the 2-year OS is approximately 18%. The prognosis of patients with CCS N0M0 is better, with a 5-year OS of 57% (74). Bianchi et al. showed that in patients with CCS and the presence of sarcoma foci in locoregional lymph nodes, 2-year OS was 40%. However, patients with lung metastases did not survive for 2 years (75). Due to the distinctive predilection of CCS for lymphogenous dissemination, it is suggested to perform SLNB in such patients, possibly followed by LND. This is a way to possibly improve the poor prognosis of patients, especially in combination with nonsurgical oncological treatment methods (75).

### 2.4 Angiosarcoma

Angiosarcoma is a rare sarcoma subtype, accounting for approximately 3% of STS (76). It shows a predilection for the location of the primary tumor within the skin (about 50% of cases); in most cases, it is the scalp. Due to the initiation of a neoplastic transformation in the component of blood and lymphatic vessels, angiosarcoma is considered a tumor with a high tendency to metastasize. Secondary foci in the lymph nodes are found in 10%–40% of patients with angiosarcoma (45, 77–79). Multiple organ metastases are often found at the time of diagnosis, which worsens the prognosis (80). Chan et al. describe the frequency of metastases to regional lymph nodes at 23% (35 patients) out of 150 patients analyzed with angiosarcoma. On the other hand, 39% (59 patients) of the patients had metastatic tumor foci found in distant organs (78). Due to the aggressiveness of this malignancy, its 5-year OS ranges from 10% to 30% (81). Furthermore, Keung et al. showed that 5-year OS for patients with N1M0 and N0M1 angiosarcoma is approximately 17% and 6%, respectively. In turn, patients with N1M1 angiosarcoma have a 5-year OS of approximately 20% (74). Kang et al. showed that almost all patients with N1M0 angiosarcoma developed distant organ metastases within half a year of observing the involvement of regional lymph nodes (82). Furthermore, Behranwala et al. showed a difference in 1-year OS in patients with angiosarcoma depending on synchronous or metachronous involvement of regional lymph nodes. The 1-year OS in the groups of patients mentioned above was 68% and 94%, respectively (83). Therefore, performing SLNB and possible LND in the early stage of sarcoma would help stop its development. The small number of patients with angiosarcoma makes it difficult to conduct reliable clinical trials.

### 2.5 Synovial sarcoma (SS)

SS is one of clinical practice’s most common STS subtypes (84). It is recognized that synovial sarcoma is one of the subtypes of sarcoma with an increased tendency to metastasize through the lymphatic vessels. Metastases at diagnosis are found in only about 5% of patients (85). However, a characteristic feature of SS is the late spread of sarcoma, usually 5 years after the diagnosis of the disease (86). Most secondary foci are found in the lungs (85% of secondary lesions), where neoplastic cells probably arrived by hematogenous dissemination (87). Lymph nodes are SS metastases’ second most common site (88). However, SS does not distinguish it from other STS with an increased predilection for lymphogenous metastases (89). The frequency of lymphogenic dissemination among patients with SS oscillates at 4%–10% (89–91). For example, Jacobs et al. conducted a retrospective analysis of a database of 885 patients diagnosed with synovial sarcoma. Lymph node metastases were present in 4.2% (37 patients) of the patients (89). Wright et al. in a meta-analysis showed that in the SS group, 6% of them (2 out of 34 people) had sarcoma cells detected in SLNB (30). In turn, Brady et al. in a retrospective cohort study of 1,550 patients with various subtypes of sarcomas showed that people with synovial-like sarcoma constituted 23% of all patients with sarcomas (360 of 1,550 patients) (66). The 5-year OS in the entire group of patients with this particular subtype of sarcoma was 84%, those without sarcoma cells in SLNB had a 5-year OS of 84%, and in patients with identified foci of synovial-like sarcoma in SLNB it was 33%. The 10-year OS in the individual analyzed patient groups was 76%, 84%, and 0%, respectively (66). It does not seem reasonable to consider the use of SLNB and LND in the diagnostic and therapeutic process of patients with SS (42, 45, 67, 92).

## 3 Sentinel lymph node biopsy in sarcomas

### 3.1 Technical aspects of SLNB

SLNB is usually performed as one of the components of surgical resection of the primary malignant tumor. All this is preceded by a biopsy of an oncologically suspicious lesion as part of the diagnostic procedure (93, 94). Histopathological confirmation of a malignant tumor allows assessing its predilection for lymphogenous metastases. In turn, the analysis of SLNB biological material allows one to determine the stage of the disease and implement effective treatment, including the ultimate radicality of surgical resection, LND (27). Although SLNB is a minimally invasive procedure, there is a risk of complications, although much lower than in the case of LND (95, 96). However, these are complications similar to those associated with LND (see Section 4.1). Mastering the proper technique to identify and remove sentinel lymph nodes (SLNs) requires surgeon experience. Performing approximately 30–40 SLNB procedures allows the surgeon to achieve the appropriate technical skills to acquire diagnostic histopathological materials (97, 98). To maintain skill in performing SLNB, it is suggested that the surgeon performs 5 such procedures per month (99). The SLNB procedure requires the location of the sentinel lymph node, its complete excretion, and its examination for the presence of sarcoma cells (100).

Lymphatic drainage causes tracers injected around the primary tumor to pinpoint regional lymph nodes, including the SLN. This procedure is done using the blue dye method (mainly isosulfan blue solution), fluorescent indocyanine green (ICG), carbon nanoparticle suspension or the radionuclide tracking method (mainly sulfur colloid labeled with technetium Tc99m) (27). The gamma radiation of radioactive tracers accurately indicates the SLN, and a gamma detector (Geiger counter) detects even those located deep in the tissue (100). ICG fluorescence is excited by near-infrared light emitted by a special device. ICG is commonly recommended for lymph node mapping, especially in obese patients and minimally invasive procedures. It is also more sensitive to detecting sentinel nodes than the blue dye method. The blue dye method is the most common of all mapping methods. However, the ease of diffusion of this dye solution causes it to spread intertissuely beyond the lymphatic vessel network, interfering with the detection of regional lymph nodes (27, 100). A modern method using a suspension of carbon nanoparticles involves the capture of these particles by macrophages. Their movement through lymphatic vessels indicates sentinel nodes. However, carbon nanoparticles adsorb chemotherapy molecules, which affects oncological therapy (27).

STS are located in both superficial and deep tissues. Therefore, injecting a tracer preparation into the area of the primary malignant tumor involves various technical challenges. SLN mapping is usually performed using a combined method - Tc99m labeled sulfur colloid labeled with Tc99m with ICG/blue dye (100). Depending on the surgeon’s experience, SLN mapping may rely solely on blue dye to properly identify the SLN. In the case of superficial tissue neoplastic lesions, 0.5 mL of radiopharmaceutical colloid is administered intradermally in four quadrants of the area of the oncological lesion approximately 2–6 h before SLNB. The skin is then lightly massaged and scans are performed with a scintillation camera imaging gamma radiation. Once the SLN is visualized as a hot spot, its location on the overlying skin is marked. SLNs become visible 1–30 min after administration of the radiopharmaceutical. Radiation that allows their localization lasts for about 4 h (27, 100). Lymphoscintigraphy performed in this way helps to specifically identify SLN, especially when there are individually variable lymph outflow tracts, for example, in the trunk (101, 102). Then, marked lymph nodes are collected. After anesthetizing the patient, approximately 1 mL of isosulfan blue solution is administered intradermally in the tumor area on both sides. The dye flows through the lymphatic vessels in 5–10 min to the radioactive SLN. After this time, the skin is incised in a previously marked place based on previous lymphoscintigraphy. After surgical access, blue-stained lymphatic vessels and sentinel lymph nodes are visible. The SLN is also detected intraoperatively with a gamma probe. Some medical centers ignore this and only perform preoperative lymphoscintigraphy (103). Usually, 1-3 lymph nodes are taken and treated as sentinel lymph nodes. In the case of malignant tumors located in deeper tissues, appropriately selected doses of markers are administered, e.g., in breast cancer, approximately 0.5 mL of colloid labeled with Tc99m and 5 mL of isosulfan blue, respectively. Additionally, malignant tumors may be located in areas that require endoscopic or surgical access. During the procedure (endoscopic, laparoscopic, or open surgery), an appropriate marker is administered directly to the area of the oncological lesion, e.g., in the large intestine or uterus. The proximity of the injection allows small doses of preparations and rapid staining of the SLN, e.g., 1 mL of isosulfan blue stains the SLN for up to 60 s in the case of colorectal cancer. Stained SLNs are marked with clips or sutures, which allows them to be identified after the label is gradually washed out. In the case of malignant tumors located in organs with pronounced motility, such as the esophagus, the use of blue dyes is limited due to the difficulty of locating stained SLNs in peristaltically moving tissues under visual control. Potential mobilization of the esophagus could interfere with lymphatic drainage. In such a situation, lymphoscintigraphy is used intraoperative using a handheld gamma probe. When the cancerous lesion is located in a small organ with closely located stations of regional lymph nodes, e.g., in the thyroid lobe, the dye is injected directly into the malignant lesion, e.g., a nodule. Administration of the preparation peritumorally would make it difficult to isolate the SLN under visual guidance (27, 100, 104).

Histopathological material collected as part of SLNB is sent to the pathology laboratory in an unfixed form or 10% neutral buffered formalin (105–107). Then it is analyzed, most often using hematoxylin and eosin staining to identify secondary lesions of the primary tumor. Immunohistochemical staining is also used to visualize molecules characteristic of specific neoplastic cells. Based on the histopathological result, another operation is decided to remove secondary malignant tumor lesions, including LND (27, 100). However, SLNB and LND can be performed during the same procedure when the patient’s clinical condition or technically difficult access to the malignant tumor make it difficult to perform another operation (108, 109). Then, an intraoperative histopathological examination of the frozen SLN preparations is performed. However, this is associated with approximately 40% lower sensitivity to detect secondary malignant tumor foci than postoperative histopathological examination (100, 110).

### 3.2 Significance in STS

The idea of SLNB is based on the occurrence of metastases of a malignant tumor through the lymphatic vessels (111). In such a situation, the SLN biopsy result allows prediction of the patient’s prognosis and determination of the progression of the disease, which influences the choice of clinical treatment, including surgical cytoreduction of secondary lesions (e.g., LND), radiotherapy and adjuvant chemotherapy (Figure 2) (100). A negative SLNB result helps prevent the patient from receiving inappropriate oncological therapies that may induce side effects. There are no indications to perform SLNB, especially when its results will not affect further patient treatment, e.g., in the elderly or with significant comorbidities that disqualify the patient from surgical treatment (100, 112). Most STS subtypes metastasize primarily through blood vessels. In sarcoma patients secondary lesions in the lymph nodes are rare and occur in approximately 1% of these cases (20). Only about 3% of people with STS of the trunk and limbs have secondary lesions in the regional lymph nodes. In many cases, they are accompanied by distant organ metastases, and the advanced stage of the malignant tumor significantly limits the effectiveness of regional lymph node resections (74, 113). Most sarcoma subtypes are believed to have a maximum 5% probability of metastasis to the lymph nodes (23). Few show a greater predilection for lymphogenic dissemination, although this is not well proven. Differences in histopathological classifications of malignant tumors, a heterogeneous cohort of few patients, the lack of standardized guidelines for performing SLNB and therapeutic procedures between specialized medical centers, and the different end points chosen in the few scientific articles on this topic are additional limitations in conducting reliable analyzes. However, the proven diagnostic and therapeutic effectiveness of SLNB with subsequent lymphadenectomy in patients with breast cancer or melanoma encourages attempts to use these procedures in people with sarcomas (30, 42). Of the approximately 100 histological subtypes of sarcomas, rhabdomyosarcoma, angiosarcoma, clear cell sarcoma, and epithelioid sarcoma are particularly prone to lymphogenous dissemination (42, 114). The potential benefits of lymph node dissection at an early stage of the disease could be used in these subtypes of STS. In addition to the histological subtype of sarcoma, the predilection for lymph node metastasis is influenced by the size of the primary malignant tumor (above 5 cm increases the tendency for lymphogenous metastasis) and high-grade sarcoma increases lymphogenous dissemination (20, 63).

Secondary STS foci in the regional lymph nodes worsen the patient’s prognosis (N1M0), which is confirmed by the small number of currently available publications that show differences in the 5-year OS among patients with selected STS subtypes at different stages of advancement (Table 3). In a meta-analysis, Wright et al. showed that among all patients with various subtypes of sarcomas, a positive SLNB result was obtained in 12% (14 out of 114 people) cases. The median OS for positive SLNB was 5 months (1–12 months). In turn, median OS in patients without sarcoma cells observed in SLNB was 48 months (8–90 months) (30). Furthermore, Johannesmeyer et al. showed that in a group of STS patients with different histological subtypes, N1M0 patients had a five-fold worse prognosis compared to STS patients localized STS (hazards ratio = 5.1, *p* < .001) (20). In turn, Brady et al. in a retrospective cohort study of 1,550 patients with various subtypes of sarcomas showed that 5-year OS in the entire study group was 79%, in the case of a negative SLNB result it was 84%, and the detection of sarcoma cells in SLNB was correlated with 49% 5-year OS. Furthermore, 10-year OS in the analyzed patient groups mentioned above was 74%, 78% and 41%, respectively (66). Also, Andreou et al. in a retrospective cohort study observed that in the group of analyzed patients with sarcomas who underwent SLNB, approximately 13% had secondary lesions detected in lymph node biopsy (8 out of 62 people). Furthermore, those with sarcoma cells detected in SLNB had a 5-year OS of 40%, and those with negative SLNB had a 5-year OS of 74% (67). Additional research analyses showed that 1-year DMFS (distant metastasis-free survival) in people with no secondary lesions in SLNB was 80% and 5-year DMFS was 60%. The confirmation of sarcoma on SLNB in patients resulted in a 1-year DMFS of 62% and a 5-year DMFS of 37% (67). The prognostic value of SLNB results for patients with sarcomas is questionable. Multicenter prospective clinical trials are needed to develop effective diagnostic and therapeutic procedures for patients with sarcomas. Currently, the validity of SLNB in these patients is not established, and the single research studies available do not allow drawing general conclusions (42).

**TABLE 3:** The 5-year overall survival (OS) of patients with selected subtypes of soft tissue sarcomas (STS) depending on the presence of secondary foci in regional lymph nodes (N) and the presence of distant metastases (M) of the STS.

Pathological type	The 5-year OS (%) in patients with advanced stage STS			References
	N0M0	N1M0	M1 (any N)	
Rhabdomyosarcoma	80	46–65	0	(47, 52, 53)
Epithelioid sarcoma	64–90	43–50	10–12	(58, 60, 61, 66, 74)
Clear cell sarcoma	57	27	0–8	(74, 75)
Angiosarcoma	34	17	6–20	(74, 81)
Synovial sarcoma	84	33	0	(66, 85)

## 4 Lymphadenectomy in sarcomas

### 4.1 Technical aspects of LND

Confirmation of the presence of secondary STS foci due to SLNB is the basis for LND (27). LND is an extension of SLNB, removing all/part of the regional lymph nodes from the primary tumor basin. Both procedures’ techniques are similar, except that LND involves resection of more of the patient’s tissue (see Section 3.1) and is done during one or two surgical procedures. Intraoperative methods of histopathological analysis of SLNB biopsies show a lower sensitivity to detect sarcoma cells compared to more time-consuming techniques used postoperatively. However, improving intraoperative biopsy examination techniques allows more accurate results to be obtained (100, 110, 115). This results in SLNB and LND being performed in one operation. Previously performed SLNB allows the patient to be protected from the more traumatic LND procedure. However, it is currently possible to perform LND using the open method and laparoscopic and robot-assisted techniques (116, 117) that limits the damage to surrounding tissues. Moreover, in certain clinical situations, LND is performed for prophylactic purposes without prior SLNB, e.g., in gastric cancer. Such action aims to perform surgical resection of the malignant tumor foci as radically as possible and improve the patient’s prognosis without exposing him to repeated surgery (118, 119).

**FIGURE 3:**
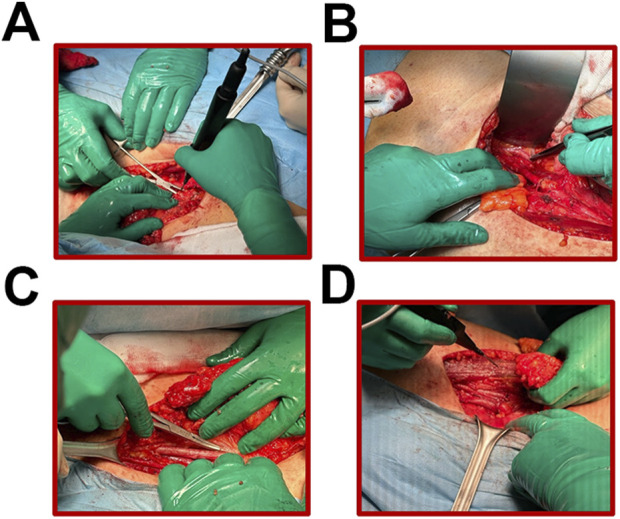
**(A–D)** Subsequent stages of lymph node dissection in the area of the obturator iliac fossa of the right groin in a patient with rhabdomyosarcoma.

LND procedures are classified according to the extent of resection and the anatomical location of the lymph nodes. The extent of surgical resection under LND may be regional or radical. This applies to some or all of the regional lymph nodes, respectively, which form the nodal station that receives lymph from the area of the malignant tumor (120, 121). In turn, the main locations of the lymph nodes subjected to dissection, depending on the location of the primary focus of the malignant lesion, include cervical, axillary, mediastinal, retroperitoneal, pelvic and inguinal LND (Figure 3) (122–127). The location of oncological lesions presents different challenges. For example, STS of the limb allows better surgical access and complete resection with a wide tissue margin and possible lymphadenectomy (9). In the case of head and neck sarcomas, extensive tissue excision during radical resection seems to be a limitation (128). The incidence of complications associated with LND varies depending on the radicality and location of the procedure performed. These include allergic reaction to the dye used, infection and dehiscence of the postoperative wound, lymphedema (e.g., of the lower extremity after inguinal LND), seroma, hematoma, damage to nearby blood vessels, nerves, and other organs, and fibrosis (127, 129, 130). Very rarely (about 300 patients have been reported worldwide), lymphangiosarcoma may occur due to probably prolonged lymphedema of the limb after dissection of many regional lymph nodes (131–133). Scaglioni et al. describe eliminating the side effects of lymph node dissection by performing lymphovenous anastomosis (LVA), which ensures postoperative lymph drainage. In addition, this publication’s authors propose using lymphatic vessel-containing tissue grafts (Lymphatic Flow Through the flap, LyFT) to reconstruct soft tissue defects after resection for oncological reasons (134). Thus, the occurrence of postoperative lymphedema is prevented. Moreover, LND is performed under general anesthesia, which is associated with anesthetic complications, for example, side effects of sedative and analgesic drugs (135).

### 4.2 Significance in STS

**FIGURE 4:**
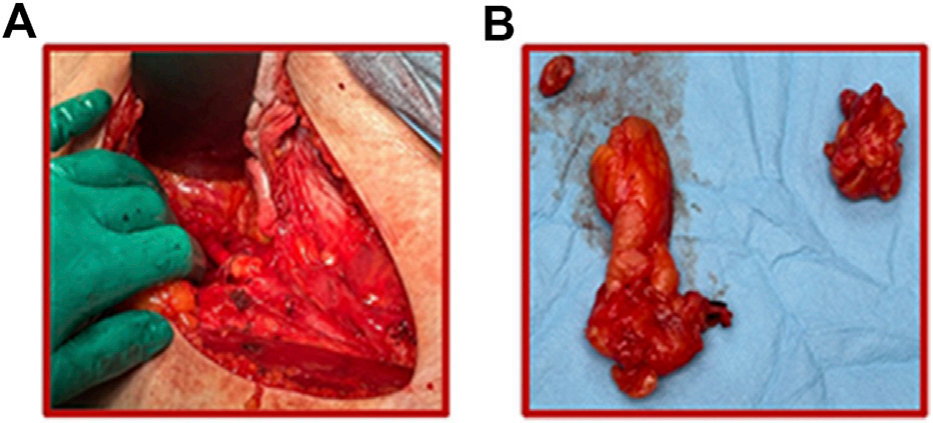
**(A)** Ilioinguinal lymphadenectomy procedure performed in the area of the obturator iliac fossa of the right groin. Operated patient with a diagnosis of rhabdomyosarcoma. **(B)** Lymph nodes isolated during this LND; upper left corner–femoral ring lymph node (lymph node of Cloquet), lower left corner–iliac lymph nodes, upper right corner–obturator lymph nodes.

The Clinical Practice Guidelines of the National Comprehensive Cancer Network (NCCN) in Oncology for STS recommend regional lymph node dissection during primary surgery in patients with node-positive sarcomas (Figure 4). In the UICC (the Union for International Cancer Control) staging system, these sarcoma patients are classified as T2-4 N1 M0 G2-3 (136). Using such treatment in a group of STS patients with various histological subtypes allowed one to improve their prognosis. Patients undergoing radical LND have been shown to have a median survival of approximately 16 months. However, the group of STS patients who did not undergo this procedure had a median survival of approximately 4 months (113). Furthermore, Sawamura et al. showed that STS patients who underwent LND had higher 1.5-year OS values than those who did not undergo LND. These values were 65% and 19%, respectively. However, the 5-year OS values in the analyzed groups of patients were similar and amounted to 30% and 19%, respectively (137). Therefore, LND improved the short-term prognosis of the patients. Some articles indicate that there is no statistically significant therapeutic benefit. Manfei et al. indicate a lack of clinical improvement among patients with uterine leiomyosarcoma undergoing LND (138). Similarly, Nasioudis et al. described the lack of benefit of LND in improving the prognosis in patients with uterine leiomyosarcoma (139). The authors do not recommend dissection of the lymph nodes in these sarcoma cases. This may be related to the low predilection of these types of sarcomas to lymphogenic metastases. However, Liu et al. indicate statistically significant benefits of prophylactic dissection of lymph nodes in STS patients with a lymphogenic tendency to sarcoma dissemination (140). Significant improvement in overall survival has been demonstrated in patients undergoing prophylactic lymphadenectomy despite the absence of tumor cells found in the surrounding lymphoid tissues (140).

Lymphadenectomy uses the basic property of malignant tumors, i.e., the ability to metastasize (141). However, there is no unequivocal evidence for lymphadenectomy based on the histological subtype of sarcoma. Sawamura et al. indicate a short-term, up to 5 years after dissection, improved survival in patients with lymphogenic rhabdomyosarcoma and improved quality of life. However, with time, the positive effect of lymphadenectomy disappears, and OS reaches values similar to those of the control group of patients (137). This surgery is considered when regional lymph node disease is suspected based on physical or radiological examination. It results in sentinel node biopsy and partial or radical lymphadenectomy. However, both a positive and a negative result of a lymph node biopsy do not necessarily determine the legitimacy of subsequent lymph node dissection (142). The explanation for this clinical approach is how malignant tumor cells spread systemically. According to the systemic model, the dissemination to lymph nodes and other organs is believed to occur independently by blood or lymphatic system (143–145).

On the other hand, Halsted’s model assumed that the dissemination of malignant tumors first occurred via the lymphatic route to regional lymph nodes. Then it spreads from them to the bloodstream or lymphatic system to other body parts (143–146). According to current knowledge, this is not true. This is confirmed by, e.g., molecular studies of malignant tumor cells taken from lymph nodes and distant organs. The sequencing of their genome points to other clonal cells being their ancestors (147–149). Therefore, they rule out the possibility that malignant tumor cells found in distant organs originated from cells found in lymph nodes. Systemic dissemination of these malignant tumor cells occurred directly through blood vessels.

Above all, the guidelines indicate the need for a comprehensive assessment of the patient’s medical history and performance status when choosing the appropriate oncological treatment (136). The result is an attempt to improve the prognosis of patients with distant metastases of STS organs by surgical cytoreduction of secondary lesions. Okiror et al. reported that resection of pulmonary metastases in selected patients with disseminated STS improves midterm survival. After this procedure, the patients had a median OS of 25.5 months and a median disease-free period of 25 months (150). Additionally, performing a repeat metastasectomy after detecting new secondary STS foci in selected patients improves their OS. Chudgar et al. showed a statistically significant prolongation of median OS in patients undergoing repeated metastasectomy compared to median OS in patients undergoing conservative oncological treatment after primary metastasectomy. The median OS values for the above groups were 44.9 months and 14 months, respectively (151). Therefore, ensuring the greatest possible radicality of surgical cytoreduction can improve the prognosis of STS patients even at an advanced stage of development. STS metastases in regional lymph nodes may result in a similarly poor prognosis for patients with distant organ metastases, such as the lungs (152, 153). Therefore, regional lymph node dissection as an element of radical surgical resection of STS may improve selected patients’ prognoses.

## 5 Summary

Radial surgical cytoreduction of neoplastic cells is effective for treating patients with malignant gastrointestinal tract, breast, or skin tumors. Removing primary and secondary malignant tumors improves the prognosis of such patients, especially when the disease is diagnosed early. The most common malignant tumors among cancer patients have a relatively well-understood pathophysiology. A proven statistically significant predilection for lymphogenous metastasis, e.g., in breast cancer, allows the use of SLNB or LND as part of the diagnostic and therapeutic procedure. Radial resection of the primary tumor and possible secondary tumors in the regional lymph nodes is a chance to achieve a good prognosis for the patient. All this suggests using a similar approach in patients with rare oncological nosological units, including STS. The small number of patients with sarcomas constitutes a significant difficulty in understanding the pathophysiology of these diseases, which could lead to the establishment of effective diagnostic and therapeutic procedures. The STS patient group includes approximately one percent of all patients with malignant neoplastic lesions. The additional histological subtypes of STS means that existing scientific articles devoted to a specific subtype of STS are often based on the analysis of several of these patients. Creating reliable, statistically significant meta-analyses or original articles makes it difficult. This results in a lack of adequate understanding of the predilection for lymphogenic dissemination, diagnosis at an early stage, or the use of effective treatment supported by evidence from randomized, controlled, multicenter clinical trials.

A literature review shows that the STS subtypes with a distinctive tendency to metastasize through lymphatic vessels are RMS, angiosarcoma, CCS and ES. Although patients with these sarcomas are characterized by hematogenous tumor spread in most situations, the reported frequency of lymphogenous metastases is higher than in other STS subtypes. However, SS is one of the majority sarcomas that do not have a predilection for dissemination through lymphatic vessels. Some scientific articles particularly emphasize the increased tendency of RMS to metastasize to regional lymph nodes in the pediatric population. Therefore, SLNB could be useful in such patients, with the possible extension of surgical resection to include LND. Other risk factors for sarcoma metastasis to lymph nodes include: grade 3 of the STS mentioned above, location of the primary tumor within the head and neck, extremes, especially the lower ones; primary tumor larger than 4 cm; male gender; age around 25. Therefore, awareness of risk factors for forming secondary foci in the lymph nodes proves helpful in diagnostic and therapeutic procedures. Especially since the presence of metastases in regional lymph nodes reduces the median OS several times compared to its value in patients with a negative SLNB result. In the case of histopathological confirmation of a sarcoma subtype known by an increased probability of metastasis to the lymph nodes, computed positron emission tomography (PET-CT) is recommended. However, no clinical studies currently confirm the validity of such a procedure. The presence of neoplastic cells in a sentinel node biopsy does not always result in distant metastases, even without lymphadenectomy. Furthermore, despite dissection of the lymph nodes based on the presence of metastases in the SLNB, the development of secondary metastatic tumors in the regional lymph nodes was observed. The presence of metastases of lymphogenic sarcomas was demonstrated in both the nodal and organ form with a negative SLNB result. The primary recommendation for clinical management is the balance of benefits and risks of the selected oncological therapy according to the clinical condition of a specific STS patient.
